# The magnitude of postpartum depression among mothers in Africa: a literature review

**DOI:** 10.11604/pamj.2020.37.89.23572

**Published:** 2020-09-25

**Authors:** Catherine Atuhaire, Laura Brennaman, Samuel Nambile Cumber, Godfrey Zari Rukundo, Grace Nambozi

**Affiliations:** 1 Faculty of Medicine, Department of Nursing, Mbarara University of Science and Technology, Mbarara, Uganda,; 2 Nova Southeastern University College of Nursing, Fort Myers Campus, 3650 Colonial Court, Fort Myers, Florida, United State of America,; 3 Centre for Health Systems Research and Development, University of the Free State, Bloemfontein, South Africa,; 4 Office of the Dean, Faculty of Health Sciences, University of the Free State, Bloemfontein, South Africa,; 5 School of Health Systems and Public Health, Faculty of Health Sciences, University of Pretoria, Pretoria, South Africa,; 6 Institute of Health and Care Sciences, the Sahlgrenska Academy at University of Gothenburg, Gothenburg, Sweden,; 7 Faculty of Medicine, Department of Psychiatry, Mbarara University of Science and Technology, Uganda

**Keywords:** Postpartum depression, prevalence, magnitude, burden, Africa

## Abstract

**Introduction:**

postpartum depression (PPD) continues to become one of the major maternal health challenges across the globe but there is a paucity of recent data on its magnitude in Africa. This study was motivated by the need to update the current magnitude of PPD in Africa based on various assessment tools.

**Methods:**

a total of 21 articles met the study criteria. Fifteen articles used the EPDS and six used other assessment tools. Postpartum depression among studies that used EPDS tool ranged from 6.9% in Morocco to 43% in Uganda and 6.1% in Uganda to 44% in Burkina Faso among studies that used other depression assessment tools. Sensitivity and specificity results of the EPDS ranged from 75%-100% and 87%-98% respectively.

**Results:**

a total of 21 articles met the study criteria. Fifteen articles used the EPDS and six used other assessment tools. Postpartum depression among studies that used EPDS tool ranged from 6.9% in Morocco to 43% in Uganda and 6.1% in Uganda to 44% in Burkina Faso among studies that used other depression assessment tools. Sensitivity and specificity results of the EPDS ranged from 75%-100% and 87%-98% respectively.

**Conclusion:**

despite the limited dearth of literature, the magnitude of PPD in Africa remains high which suggests that PPD is still a neglected illness and calls for immediate interventions. EPDS is an effective tool with high sensitivity and specify in varying study contexts.

## Introduction

Postpartum depression (PPD) continues to become one of the major maternal health challenges across the globe [1-3]. A diagnostic criterion for PPD is occurrence within six weeks to six months following childbirth and symptoms must manifest for at least two weeks. PPD is one of the most challenging complications of pregnancy due to adverse effects on the health of the mother, infant, family, and community at large [4]. Detrimental consequences of PPD include diminished mother to child bonding, childhood growth impairment and underdevelopment, infanticide, and suicide [5,6]. PPD is characterized by loss of interest in usual events, sleep challenges, feelings of sadness, fatigability, problems of appetite, and difficulty in coping with daily activities [7]. Because postpartum depression is one of the most common treatable complications of the postpartum period, it is paramount that health workers identify and manage the condition promptly and effectively.

Determining the current global PPD prevalence has remained a challenge due to use of varying assessment tools, adoption of different cut-off points for those tools, the varying cultural contexts, and the dearth of studies carried out in resource-limited environments [2]. Researchers [1-3] continue to show conflicting PPD prevalence rates. Between 2012 and 2016, the global mean prevalence for PPD was reported between 15% and 25% [1-3], a higher value than the 12% to13% reported in 2007 [8], thus suggesting an increasing PPD prevalence. PPD is more rampant by 25% in low-and middle- income countries as compared to developed countries [2,9-11]. Literature reports specific countries postpartum depression rates to be 10.1 percent in Norway [12], 8 to 10 percent in Netherlands [13,14], 3.9 to 17.6 percent in Portugal [15-17] , 8 to 12.3 percent in Sweden [18-20] while in United States of America, it is estimated at 8 to 15 percent [21-24].

In Africa, studies on the magnitude of postpartum depression remain scanty [25,26].The few existing studies estimate the magnitude to be 15 to 25 percent [27-32]. Because the prevalence of PPD is projected to be higher in Africa than the global mean prevalence [33], there is still a paucity of data available in many African countries. This lack of consistent data has compromised the development of a cumulative body of knowledge regarding the multi-cultural prevalence and magnitude of PPD [34]. This review therefore aims at summarizing and synthesizing the current magnitude of postpartum depression among mothers based on various assessment tools in Africa.

## Methods

A review of literature was undertaken from seven electronic databases which included PubMed, Google Scholar, CINAHL, Africa Bibliography, Bibliography of Africana Periodical Literature, African Journal On-Line, and PsychInfo. The time frame for the reviewed articles ranged from May 19, 1995 to May 18, 2020. The search identified a total of 120 articles amongst which only 21 research articles met the inclusion criteria. We started our search in 1995 to exhaust a wide range of synthesis about documented changes in magnitude over the years in Africa. Boolean strategy was used to search the databases. We used a combination of Medical Subject Heading (MeSH) and free-text terms to search the databases using the keywords: postpartum depression, prevalence, magnitude, burden, combined with Africa.

**Eligibility criteria**: studies were considered eligible if they were published in English, carried out within the stated time-frame, focused on the magnitude of postpartum depression in Africa, used scientific methods such as surveys, case-control, trials, prevalence and surveillance studies with sound methodological standards. These studies were further considered if they were original publications and were carried out in community and healthcare facilities. For validation studies, only articles that validated the tools of assessment were included. The review excluded articles that were not available in full text, depression assessment method not well described, and the assessment tools used were not validated.

**Data screening and extraction**: the data screening and extraction process involved removal of duplicate articles, initial screening of the titles and abstracts based on the eligibility criteria and full-text screening of selected studies. All the articles that met our inclusion criteria were retained for data extraction using an electronic standardized data extraction template designed by the team. The data extraction template was first pilot-tested on a representative sample of articles. The characteristics of studies included the authors and year of publication, the country of study, the sample size, the type of depression assessment tool, time frame of assessment and magnitude or prevalence of depression among postpartum mothers (Table 1 and Table 2). A narrative summary of the extracted data was performed to address the research aims of the review.

**Table 1:** the magnitude of postpartum depression in Africa using the EPDS as an assessment tool

SN.	Author	Country	Sample size	Assessment tool	Time frame of assessment	Magnitude
1	Hung, 2014 [45]	South Africa	249	EPDS	12 weeks	31.7%
2	McHichi Alami *et al*. 2006 [46]	Morocco	100	EPDS	12 weeks	17.0%
3	Abiodun, 2006 [47]	Nigeria	360	EPDS	6 weeks	18.6%
4	Adewuya *et al*. 2005 [27]	Nigeria	128	EPDS	6 weeks	14.6%
5	Stellenberg, 2015 [48]	South Africa	159	EPDS	6 weeks	50.3%
6	Uwakwe, 2003 [49]	Nigeria	225	EPDS	6-8 weeks	10.7%
7	Agoub *et al*., 2005 [50]	Morocco	144	EPDS	6 weeks	6.9%
8	Kakyo *et al*.2012 [29]	Uganda	202	EPDS	< 12 weeks	43%
9	Rogathi, 2017 [51]	Tanzania	1013	EPDS	6 weeks	12%
10	Chibanda, 2010 [28]	Zimbabwe	210	EPDS	6 weeks	33%
11	Chinawa, 2016 [52]	Nigeria	214	EPDS	6 weeks	22.9%
12	Khalifa, 2015 [30]	Sudan	238	EPDS	12 weeks	9.2%
13	Madeghe, 2016 [53]	Kenya	200	EPDS	6 weeks	13%
14	Ongeri, 2018 [54]	Kenya	171	EPDS	6-10 weeks	18.7%
15	Abadiga, 2019 [55]	Ethiopia	287	EPDS	< 12 weeks	20.9%

**Table 2:** the magnitude of postpartum depression in Africa using other assessment tools

SN	Author	Country	Sample size	Assessment tool	Time frame of assessment	Magnitude
1	Anokye, 2018 [56]	Ghana	212	PHQ-9	12 months	7%
2	Baggaley, 2007[57]	**Burkino Faso**	61	K10	12 weeks	44%
3	Adewuya & Afolabi, 2005 [58]	Nigeria	480	ZSDS	12 weeks	13.1%
4	Nakku, 2006 [26]	Uganda	544	SRQ-25	6 weeks	6.1%
5	Toru, 2018 [59]	Ethiopia	456	PHQ-9	12 months	22.9%
6	Odinka, 2019 [60]	Nigeria	424	HADS	6-14 weeks	33.3%

## Results

**Figure 1:**
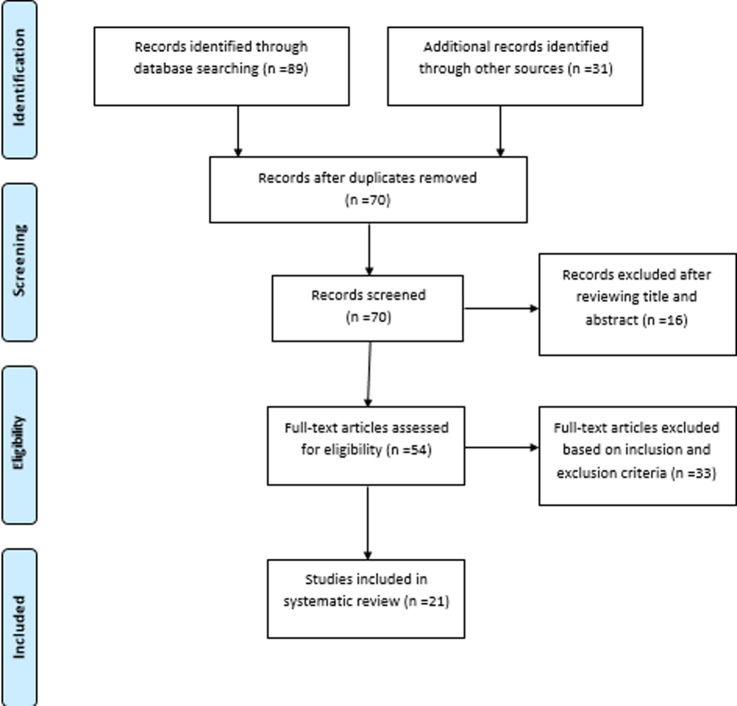
flow diagram of the article selection procedure for articles published between the year 1995 and 2020 on the magnitude of postpartum depression among mothers in Africa

The literature search carried out on the magnitude of postpartum depression among mothers in Africa identified 21 studies. These included 15 articles on magnitude of postpartum depression among mothers in Africa using the EPDS, six articles on magnitude of postpartum depression among mothers in Africa using other PPD assessment tools and four of the articles that used the EPDS tool validated it in the African local languages and cultures as shown in Table 3. The flow diagram as shown in Figure 1 presents the inclusion and exclusion process.

**Table 3:** studies validating the EPDS in local African languages for detection of postpartum depression in Africa

SN.	Author	Country	Language	Sample size	Diagnostic instrument Diagnostic criteria	Sensitivity (%) at different EPDS cut-off points		Specificity (%) at different EPDS cut-off points	
						9/10	12/13	9/10	12/13
1	Adewuya *et al*.2005[27]	Nigeria	Yoruba	876	DSM-III	-	100	-	98
2	Chibanda et al. 2010 [28]	Zimbabwe	Shona	210	DSM-IV	-	88	-	87
3	Uwakwe, 2003 [49]	Nigeria	Igbo	225	ICD-10	63	75	97	98
4	Agoub et al. 2005 [50]	Morocco	Arabic	144	MINI	-	92	-	96

Fifteen articles that used EPDS in determining the magnitude of postpartum depression in nine African countries were reviewed. Table 1 shows that the postpartum depression ranged between 6.9% to 43% percent. Findings show that the magnitude of postpartum depression in South Africa (31.7-39.6%) [35,36], Morocco (6.9-14%) [37,38], Nigeria (10.7-22.9%) [28,39-41], Uganda (43%) [27], Tanzania (12%) [42], Zimbabwe (33%) [30], Sudan (9.2%) [32], Kenya (13-18.7%) [43,44] and (19.9%) participants in Ethiopia [45]. Two studies were carried out in South Africa, Morocco, Kenya and four studies qualified to be included in Nigeria. This review targeted articles that studied postpartum mothers between six and twelve weeks following child birth. Majority of these studies ten (10) assessed PPD at six weeks whereas five (5) studies detected it at less than twelve weeks postpartum. These studies identified all postpartum mothers aged 17-49 years, who visited the health facility for routine immunization of the infants and postpartum care, during the data collection period.

Table 2 shows that there was no serious variance in the magnitude as reported in table 1 where the authors used the EPDS in detecting PPD. The review covered six studies that used other tools of assessment. These included 2 Patient Health Questionnaire (PHQ-9) [46,47], 1 Kessler (K10) [48], 1 Zung´s Self Rating Depression Scale (ZSDS) [49], 1 Self Reporting Questionnaire (SRQ-25) [25], and 1 study used Hospital Anxiety and Depression Scale (HADS) [50]. Results show that postpartum depression ranged between 6.1% and 44%. Country specific prevalence reported were Ghana (7%) [46], Burkino Faso (44%) [48], Nigeria (13.1-33.3%) [49,50], Uganda (6.1%) [25] and (22.9%) participants in Ethiopia [47]. All these countries had one study reporting the prevalence apart from Nigeria that had two studies included in this review. These six studies targeted all postpartum mothers that visited the health facility at the time of data collection and had delivered up to twelve months as opposed to the studies that detected PPD using the EPDS between six and twelve weeks following child birth.

Some of the review studies translated and validated the EPDS tool to the local language. Such studies were scanty and as indicated in Table 3 include four articles reviewed from three countries namely: Nigeria in Igbo and Yoruba, Zimbabwe in Shona and Morocco in Arabic language. One (1) of the studies used EPDS cut-offs of 9/10 and 12/13 cutoff for sensitivity and specificity [40] while the other three (3) studies used cutoff point of only 12/13 for both the sensitivity and specificity [28,30,38]. All the studies reported high sensitivity and specificity rates of 75%-100% and 87%-98% of 12/13.

## Discussion

Postpartum Depression has been established by several studies [2,51,52] to have significant consequences for women´s health outcomes and their quality of life. Additional impacts of PPD include maternal bonding, child health and development. The prevalence of PPD varies among countries, regions, and socioeconomic settings [2,24,51,52]. Many studies have shown the prevalence of postpartum depression around the world, but there is no universal prevalence rate that the literature indicates. The prevalence rates are higher in low and middle- income countries as compared to developed countries [1,9-11,53]. Most of these studies have used western screening tools for PPD assessment without validating them for local contexts yet PPD may be perceived in different ways across cultures. Secondly, most of the studies reviewed used self-report measures which may lead some communities in over-estimating or underestimating according to their beliefs, perceptions, and social contexts. It would be more accurate if the mothers are screened using a self-reporting questionnaire augmented with further clinical diagnostic evaluation to confirm the validity of the screening tools. Some researchers have pointed out that PPD prevalence peaks between six and twelve weeks if the EPDS is used but may increase to twelve months if other tools of assessment like the PHQ-9 are used [5]. This therefore emphasizes the need to consistently consider the uniform time frame while assessing for PPD.

In developed countries, many studies examining the prevalence of postpartum depression using the EPDS show low prevalence rates compared to low and middle-income countries, which have prevalence variations [22,54-57]. The PPD rate is estimated to range from 3.9 to 17.6 percent in five countries hence: Norway, Netherlands, Portugal, Sweden and the United States of America [12-24]. The intense attention to PPD in developed countries may have contributed to awareness and policy interventions to address PPD. This could explain the lower prevalence rate than in low and middle income countries. Secondly, the available screening tools have been developed in the context of western cultures and thus reflect the cultural perceptions and understanding in those settings. The discrepancies point to the urgency of a locally validated tool to screen for PPD. The few existing studies estimate higher prevalence of PPD in the African countries. The paucity of consistent PPD prevalence data in African countries [25,26] may be due to the in-availability of screening tools developed within the context of local cultures that reflect the African cultural perceptions and understanding. Other prevalence studies that were carried out were excluded because they were not specifically screening for PPD but were directed to high risk categories like HIV infected women, pregnant women, pregnant women with eclampsia and mothers of children with malnutrition [27,58-60].

## Conclusion

In conclusion, the literature review shows that primary studies examining the magnitude of PPD are very few which means the condition is under reported. The magnitude of PPD in Africa using EPDS ranges from 6.9% to 50.3% and 6.1% to 47.7% among studies that used other tools. EPDS tool is an effective tool for assessing PPD as results showed sensitivity and specificity results ranged from 75%-100% and 75%-98% respectively in different settings. **Recommendations**: 1) African countries need to consider postpartum depression as a public health problem that is neglected among postpartum mothers. 2) Need to determine the actual prevalence of postpartum depression using a validated Edinburgh Postnatal Depression Scale (EDPS) against a clinical diagnosis based on a DSM-V, MINI or ICD-11 among the African population. 3) The actual burden of PPD will encourage early screening, diagnosis and treatment of postpartum depression as an integral component of maternal postpartum care in Africa.

### What is known about this topic

- The articles reviewed have provided great insights into what is already known about the magnitude of PPD and the existing limitations and knowledge gaps have also been identified;
- Validation of screening tools into local contexts is very important in appropriately determining the actual prevalence of PPD among mothers;
- The use of western developed standardized screening tools may not be appropriate for application across variable cultural groups requiring researchers to be culturally sensitive; the Edinburgh Postnatal Depression Scale is one of the most reliable and simple tools for screening PPD among mothers with a high level of acceptability.

### What this study adds

- The review of the literature has found that there are limited studies carried out to determine the magnitude of postpartum depression in Africa;
- Available studies on the prevalence of PPD have given contradicting variable results. These differences are attributed to the different tools used, varying cutoff points, timing, and cultural settings.
